# EEG power spectrum analysis for Tunisian schizophrenic patients

**DOI:** 10.1192/j.eurpsy.2023.2283

**Published:** 2023-07-19

**Authors:** M. Mnif, N. Smaoui, L. Triki, J. Donia, R. Feki, I. Gassara, S. Omri, M. Maalej, N. Charfi, L. Zouari, J. Ben Thabeut, K. Masmoudi, M. Maalej

**Affiliations:** 1 Psychiatry C department, Hedi Chakeur Hospital; 2 Functional exploration department, Hbib Borguiba Hospital, Sfax, Tunisia

## Abstract

**Introduction:**

Schizophrenia (SCZ) is a common and disabling psychiatric condition. Its diagnosis is entirely clinical. Many researchers have looked into electroencephalogram (EEG) power spectrum analysis for SCZ to find specific abnormalities.

**Objectives:**

The objective of this study was to analyze the EEG of patients with SCZ using power spectral density and to compare them with those of healthy controls, in order to look for specificities.

**Methods:**

This was a cross-sectional, descriptive, and analytical case-control study conducted with patients followed for SCZ in the psychiatry "C" department at the Hedi Chaker hospital in Sfax. Healthy controls were included. Patients were assessed by the Positive and Negative schizophrenia scale (PANSS). All participants benefited from an EEG at rest condition at the service of the functional exploration at the Habib Bourguiba hospital in Sfax. We have measured the powers of each band using Welch power spectral density method called absolute power (AP). Statistical analyses were carried out.

**Results:**

Fifteen schizophrenics and 15 controls, all male, were included. The average age of schizophrenics and healthy controls was 40 years ±12.72 years and 47.93 ± 15.61 years respectively. There were no significant differences in age between patients and controls. Schizophrenics had a mean PANSS of 64.6±22.7.

At the quantitative EEG, differences appeared to be insignificant. There was an overall decrease in AP for the alpha band particularly in the parietal and occipital lobes in schizophrenics (53,16 ± 48,83 μV2 and 75,17 ± 56,28 μV2 respectively) compared to controls (335,15 ± 994,73 μV2 and 400,24 ± 1109,95 μV2 respectively). There was also an overall decrease in AP for the different frequency bands in schizophrenics compared to controls. However, values persisted high in the temporal lobe for all frequency bands.

**Conclusions:**

In conclusion, this decrease in AP for the alpha band in the parietal and occipital lobes in schizophrenics can be a sensitive biomarker for diagnosing SCZ.

**Disclosure of Interest:**

None Declared

